# Supplementary material for: Photodynamic Therapy Using Talaporfin Sodium for Local Failure after Chemoradiotherapy or Radiotherapy for Esophageal Cancer: A Single Center Experience
Source: J Clin Med. 2020 May 17;9(5):1509. doi: 10.3390/jcm9051509 (PMC7290876; doi:10.3390/jcm9051509)
Supplement: Supplementary file 1 [file jcm-09-01509-s001.pdf]

Supplemental material

Table S1. Patients' data with esophageal stricture after PDT: baseline characteristics at prior treatment and results of PDT

Patients			Baseline characteristics at prior treatment					Results of PDT									
Case	Sex	Age	Tumor location	Histological type	cStage at pretreatment	Prior treatment (regimen)	Total dose of radiotherapy (Gy)	Tumor status	Invasion depth	Circumference of the lesion	Longitudinal lesion length (cm)	Interval between CRT and PDT (months)	Total dose of irradiation (J)	Local efficacy	Period until onset of stenosis (months)	Required number of EBD	Outcome
1	F	75	Middle	SCC	T3N0M0, cStage II	CRT CDDP+5-FU	70	Recurrence	SM	1/2	4	132	700	L-CR	4	7	Survival: 36 months after PDT
2	M	69	Lower	SCC	T4N0M0, cStage IV_A	CRT CDDP+5-FU	60	Recurrence	SM	1/3	2	192	650	L-CR	5	4	Survival: 32 months after PDT
3	F	63	Middle	SCC	T4N0M0, cStage IV_A	CRT CDDP+5-FU	60	Recurrence	M	1/3	2	120	400	L-CR	9	2	Survival: 29 months after PDT
4	F	72	Middle	SCC	T1bN0M0, cStage I	CRT CDDP+5-FU	60	Recurrence	SM	1/4	1	7	400	L-CR	3	5	Survival: 21 months after PDT
5	M	69	Upper	SCC	T1bN0M0, cStage I	CRT CDDP+5-FU	60	Residual	SM	1	2	8	1100*	L-CR	1	10	Survival: 13 months after PDT

PDT, photodynamic therapy; SCC, squamous cell carcinoma; CRT, chemoradiotherapy; CDDP, cisplatin; CDGP, nedaplatin; L-CR, local complete response, EBD; endoscopic balloon dilation

* Total dose of three PDTs
